# Fetal Calcium Regulates Branching Morphogenesis in the Developing Human and Mouse Lung: Involvement of Voltage-Gated Calcium Channels

**DOI:** 10.1371/journal.pone.0080294

**Published:** 2013-11-25

**Authors:** Sarah C. Brennan, Brenda A. Finney, Maria Lazarou, Anne E. Rosser, Caroline Scherf, Dirk Adriaensen, Paul J. Kemp, Daniela Riccardi

**Affiliations:** 1 Cardiff School of Biosciences, Cardiff University, Cardiff, United Kingdom; 2 Center for Cardiovascular Sciences, Institute for Biomedical Research, University of Birmingham, Edgbaston, United Kingdom; 3 Cardiff and Vale University Health Board, Llandough Hospital, Cardiff, United Kingdom; 4 Laboratory of Cell Biology and Histology, Department of Veterinary Sciences, University of Antwerp, Antwerp, Belgium; Children's Hospital Los Angeles, United States of America

## Abstract

Airway branching morphogenesis *in utero* is essential for optimal postnatal lung function. In the fetus, branching morphogenesis occurs during the pseudoglandular stage (weeks 9–17 of human gestation, embryonic days (E)11.5–16.5 in mouse) in a hypercalcaemic environment (∼1.7 in the fetus vs. ∼1.1–1.3 mM for an adult). Previously we have shown that fetal hypercalcemia exerts an inhibitory brake on branching morphogenesis via the calcium-sensing receptor. In addition, earlier studies have shown that nifedipine, a selective blocker of L-type voltage-gated Ca^2+^ channels (VGCC), inhibits fetal lung growth, suggesting a role for VGCC in lung development. The aim of this work was to investigate the expression of VGCC in the pseudoglandular human and mouse lung, and their role in branching morphogenesis. Expression of L-type (Ca_V_1.2 and Ca_V_1.3), P/Q type (Ca_V_2.1), N-type (Ca_V_2.2), R-type (Ca_V_2.3), and T-type (Ca_V_3.2 and Ca_V_3.3) VGCC was investigated in paraffin sections from week 9 human fetal lungs and E12.5 mouse embryos. Here we show, for the first time, that Ca_v_1.2 and Ca_v_1.3 are expressed in both the smooth muscle and epithelium of the developing human and mouse lung. Additionally, Ca_v_2.3 was expressed in the lung epithelium of both species. Incubating E12.5 mouse lung rudiments in the presence of nifedipine doubled the amount of branching, an effect which was partly mimicked by the Ca_v_2.3 inhibitor, SNX-482. Direct measurements of changes in epithelial cell membrane potential, using the voltage-sensitive fluorescent dye DiSBAC_2_(3), demonstrated that cyclic depolarisations occur within the developing epithelium and coincide with rhythmic occlusions of the lumen, driven by the naturally occurring airway peristalsis. We conclude that VGCC are expressed and functional in the fetal human and mouse lung, where they play a role in branching morphogenesis. Furthermore, rhythmic epithelial depolarisations evoked by airway peristalsis would allow for branching to match growth and distension within the developing lung.

## Introduction

Efficient gas exchange in the postnatal lung requires optimal formation of the bronchial tree in the fetus [1], [2]. Lung development begins around embryonic day (E)9.5 in mice and week 3–4 post-conception in humans and comprises of five stages [1]. During the *pseudoglandular* stage (E11.5 - 16.5 in mice, weeks 5 – 17 in humans), the developing epithelium grows into the mesenchyme where it undergoes stereotypic branching and budding, leading to airway formation [3]. Secretion of lung fluid into the lumen throughout gestation generates the distending pressure for normal growth [4]. Excessive or reduced lumen distension [5], [6] yields hyperplastic or hypoplastic lungs, respectively [1]. At the same time, rhythmic peristaltic contractions causing transient and cyclic airway occlusions develop, persisting throughout gestation [1], [7], [8] and create the mechanical stimulus that propels the fluid secreted into the airway lumen towards the tips of the developing lung [8], [9]. Spontaneously occurring, cyclic intracellular calcium waves present in airway smooth muscle cells immediately precede the peristaltic waves. While it is well established that these airway smooth muscle waves require the presence of both intracellular calcium ions (Ca^2+^_i_) and extracellular calcium ions (Ca^2+^_o_), a firm link between generation of airway smooth muscle waves, airway peristalsis and lung development has never been established.

Development of the fetal lung occurs in a relatively hypercalcaemic environment, as free ionized extracellular calcium concentration ([Ca^2+^]_o_) is approximately 1.6–1.7 mM in both humans and mice [10]. This level is significantly higher than the adult concentration of 1.1–1.3 mM, and this relative fetal hypercalcaemia is maintained irrespectively of maternal [Ca^2+^]_o_ [11] and is thought to be required for skeletal accrual of Ca^2+^ by the fetus [10]. In addition to fulfilling a role in optimal bone formation, we have demonstrated that this relative fetal hypercalcaemia regulates organ development *in utero* [12], [13]. Indeed, using a lung culture model, we showed previously that [Ca^2+^]_o_ similar to that seen in gestation (*i.e*. 1.7 mM) suppresses lung branching morphogenesis and cellular proliferation while increasing fluid secretion [12]. In contrast, [Ca^2+^]_o_ comparable to those seen in the adult (*i.e*. 1.0 – 1.2 mM) induce the opposite effect - an increase in lung branching morphogenesis and a suppression of fluid secretion. These effects of Ca^2+^_o_ on lung branching and fluid secretion are mediated through the extracellular calcium-sensing receptor (CaSR) [12], [14], a G protein-coupled receptor whose expression, in the developing mouse and human lung, is confined to the pseudoglandular phase [12], [15]–[17]. In addition to CaSR-mediated effects on lung development, previous studies have demonstrated the importance of L-type Ca^2+^ channels in lung development. Indeed, treatment of pseudoglandular mouse lung rudiments with nifedipine prevents airway peristalsis and causes lung hypoplasia [18], [19]. These results suggest that, in addition to their ability to suppress branching morphogenesis through the CaSR, calcium ions regulate lung growth through voltage-gated, L-type calcium channels. Yet, the identity of the voltage-gated calcium channels (VGCC) in the fetal lung and the existence of a functional link with lung growth are unknown.

In this study, we sought to determine if, in addition to its established effects through the CaSR, the hypercalcaemic environment of the fetus could regulate branching morphogenesis through activation of VGCC present in the developing lung. Initially, we determined the expression of a variety of L, P/Q, N, R and T-type VGCC by performing immunohistochemistry on serial sections of human lungs at 9 weeks post-conception and of mice at an equivalent stage of gestation (*i.e*., E12.5). Subsequently, we assessed the effects of selective inhibitors of VGCC on branching morphogenesis in pseudoglandular mouse lung rudiments cultured in chemically defined, serum-free conditions. Finally, we tested the possibility that these channels could be activated using combined electrophysiological and biophysical methods.

## Methods

### Ethical approval

Wild type C57BL/6 were housed conventionally with 12 h light:dark cycle with free access to food and water. All animal procedures were carried out in the UK and were approved by the UK Home Office and carried out in accordance with the Animal (Scientific Procedures) Act 1986.

Human fetal tissue was collected following the guidelines of the Polkinghorne [20] and Department of Health [21] reports and with Bro Taf Local Research Ethics Committee approval. Full written consent was obtained from the maternal donor, following consent for the termination, as part of the Medical Research Council (UK)-sponsored, South Wales initiative for transplantation (SWIFT) program. Human fetal lung tissue was obtained from ethically-consented maternal donor medical termination at 9 week of pregnancy. Gestational age was first assessed by ultrasound and confirmed using fetal morphometric parameters after therapeutic abortion.

### Immunohistochemistry

Human fetal lungs (9 week post-conception) or mouse E12.5 embryos were fixed in 4% paraformaldehyde overnight and subsequently embedded in paraffin. 5 µm thick, paraffin-embedded sections were deparaffinised in xylene and rehydrated using a decreasing alcohol-water series (100%, 90%, 75% ethanol), followed by washes in distilled water. After antigen retrieval in citrate buffer, non-specific staining was prevented by incubating the slides with blocking solution (phosphate-buffered saline added with 1% bovine serum albumin and 5% Sea Block – Thermo Scientific, Cramlington, U.K.) for 1 h at room temperature. Primary antibodies against L-type (Ca_V_1.2 and Ca_V_1.3), P/Q type (Ca_V_2.1), N-type (Ca_V_2.2) R-type (Ca_V_2.3) and T-type (Ca_V_3.2 and Ca_V_3.3) Ca^2+^ channels were diluted in blocking solution (1/100 for mouse E12.5 whole embryos, 1/50 for human fetal lungs) and applied to the slide for 12 – 16h at room temperature. Primary antibody suppliers and dilutions used were [22]:

Cav1.2 - rabbit polyclonal (Alomone Labs; mouse: 1/100, human: 1/50)

Cav1.3 - rabbit polyclonal (Alomone Labs; mouse: 1/100, human: 1/50)

Cav2.1 - rabbit polyclonal (Alomone Labs; mouse: 1/100, human: 1/100)

Cav2.2 - rabbit polyclonal (Millipore; mouse: 1/100, human: 1/100)

Cav2.3 - rabbit polyclonal (Abcam; mouse: 1/100, human: 1/100)

Cav3.2 - goat polyclonal (N-18) (Santa Cruz; mouse: 1/100, human:1/100)

Cav3.3 - goat polyclonal (N-20) (Santa Cruz; mouse: 1/100, human:1/100)

Secondary antibody suppliers and dilutions were: goat anti-rabbit horse radish peroxidase (HRP) (Cav 1.2, 1.3, 2.1, 2.2, and 2.3; DAKO, Ely, U.K.; 1:200 for both mouse and human) or donkey anti-goat HRP (Cav 3.2 and 3.3; Abcam; 1:200 for both mouse and human).

The secondary, HRP-conjugated antibodies were applied for 1 h at room temperature. Antigen-antibody binding was visualized with diaminobenzidine (Sigma-Aldrich), after which the slides were counterstained with haematoxylin. Negative controls were carried out through substitution of primary antibodies with rabbit serum. The slides were dehydrated using an increasing alcohol series (30s in 75% ethanol, 4 min in 100% ethanol) and finally cleared in xylene before being mounted using DPX mounting medium (Depex-Polystyrene in xylene, Timstar laboratory Suppliers, Ltd, Marshfield Bank, U.K.). Slides were left to dry overnight and then photographed using a microscope attached to an Infinity 2-2C CCD camera (Lumenera, Ottawa, Canada) and/or scanned using a slide scanner (MIRAX SCAN, Carl Zeiss MicroImaging GmbH, Göttingen, Germany).

### Measurements of lung branching morphogenesis

Lungs explanted from E12.5 mice were cultured for 48 h according to previously published protocols [12], [23]–[25]. Images were captured at 0 and 48 h with a dissecting microscope equipped with a digital camera (Leica Microsystems, Milton Keynes, UK). Branching morphogenesis was quantified and is expressed as an ‘increase in branching after 48 h (%)’, determined using the following equation: (Branches_48h_ – Branches_0h_)/(Branches_0h_) x 100. The [Ca^2+^]_o_ in the DMEM-F12 medium employed for these experiments was 1.05 mM, representative for the adult, “low Ca^2+^_o_” conditions. For the fetal, “high Ca^2+^_o_” conditions, [Ca^2+^]_o_ in the DMEM-F12 was increased from 1.05 mM [Ca^2+^]_o_ to 1.70 mM [Ca^2+^]_o_ using 1 M CaCl_2_ (Sigma-Aldrich, Gillingham, UK). The dihydropyridine nifedipine (Sigma-Aldrich, Gillingham, UK) and the tarantula toxin SNX-482 (Tocris Bioscience, Bristol, UK) were used as Ca^2+^ channel blockers. Nifedipine was dissolved in ethanol (0.001% final concentration) while SNX-482 was dissolved in DMSO (0.001% final concentration). Vehicle control experiments were performed by adding the equivalent amount of ethanol or DMSO to the lung cultures. Data are presented as the mean ± the standard error of the mean (SEM) from multiple pooled experiments. Significance was determined using a one-way ANOVA with Tuckey’s *post hoc* test using GraphPad Prism 6.01 software (GraphPad Software, La Jolla, CA, USA).

### Loading and visualization of the voltage-sensitive probe - DiSBAC_2_(3)

Lungs explanted from E12.5 from C57BL/6 mice were attached to filters, visualized using an Olympus CK41 inverted microscope (Olympus, Southall, U.K.) and secured using a slice anchor (Warner Instruments, Hamden, CT, USA). A 5 MΩ borosilicate glass electrode (World Precision Instruments, Stevenage, U.K.) was filled with a solution containing 5 µM DiSBAC_2_(3) (Invitrogen, Paisley, U.K.) in 0.4% trypan blue/0.85% saline solution (Invitrogen). Electrodes were slowed pushed into a terminal lumen whilst maintaining positive pressure, with the presence of trypan blue in the lumen being indicative of successful access to the lumen. Slow, continuous positive pressure allowed entry of the loading solutions to sections of the lumen, and lungs were then incubated for 30 – 45 min at 37°C. DiSBAC_2_(3) fluorescence was visualized using a Cell Map IC confocal laser-scanning microscope (BioRad, Hemel Hempstead, U.K.), coupled with a water-immersion BX50WI and a UM Plan Fl 10x/0.30 W objective (both from Olympus, Southend-on-Sea, U.K.). Lungs were secured using a slice anchor in a 35 mm cell culture dish in 5 mL of solution containing in (mM): 135 NaCl, 5 KCl, 1.23 MgCl_2_, 1.0 mM CaCl_2_, 5 HEPES, 10 glucose, pH 7.4. DiSBAC_2_(3) was excited at 532 nm and images were collected using a 560 nm long pass filter, by the direct method. Images were taken at 6s intervals over a ten minute time period at room temperature and then processed and analysed with the software programs LaserSharp 2000 (Carl Zeiss, Cambridge, U.K.) and ImageJ 1.46r (National Institute of Health). After 5 minutes, the KCl concentration in the extracellular solution was increased to 50 mM. For DiSBAC_2_(3) fluorescence intensity, regions of interest were selected and then followed in either the vertical or horizontal plane to correct for lung movement and contractions. Average pixel intensity was measured using ImageJ, with increases in fluorescence indicative of depolarization of the membrane, before being plotted using GraphPad Prism 6.0 (GraphPad).

## Results

### Human and mouse pseudoglandular lungs express L-type and R-type Ca^2+^ channels

**Figure 1:**
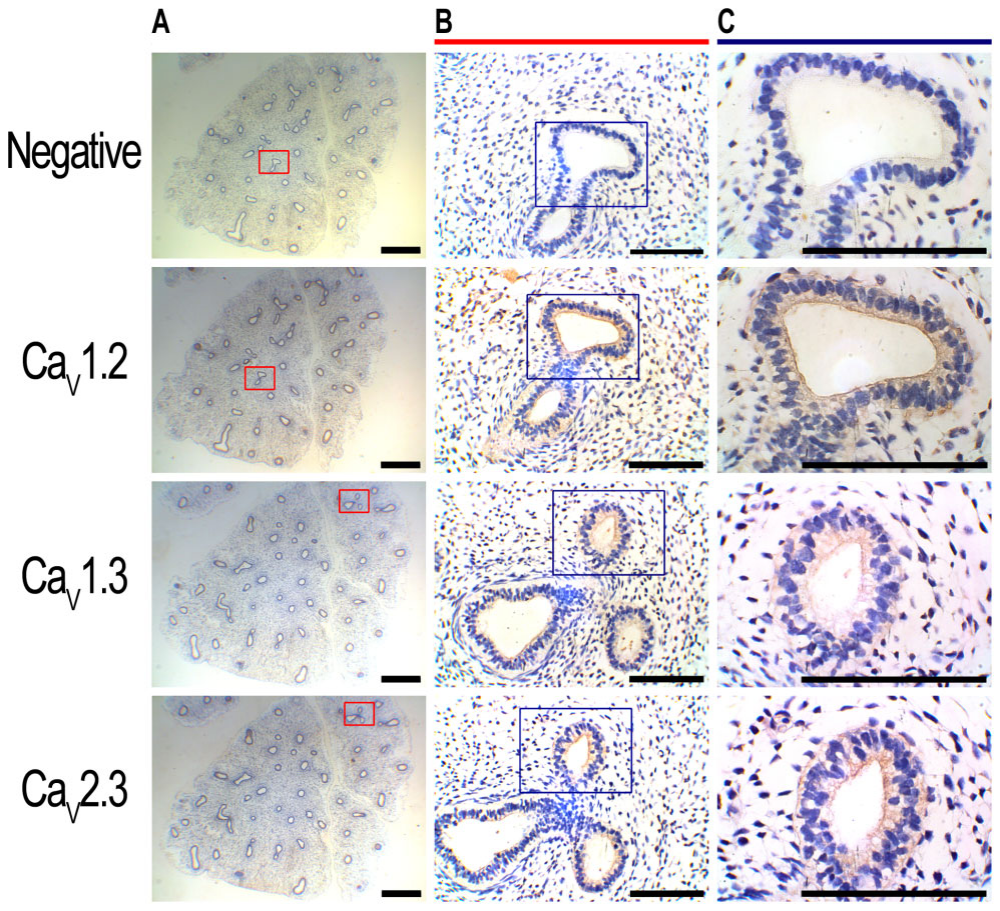
The L-type calcium channels, Ca_v_1.2 and Ca_v_1.3, and the R-type calcium channel, Ca_v_2.3, are expressed in 9 week human developing lungs. Paraffin-embedded, 5 µm-thick serial sections from an 9 week human fetal lung were dewaxed and used for immunohistochemistry. A: Expression of Ca_v_1.2, Ca_v_1.3 and Ca_v_2.3 in the lung epithelium, visualised using DAB (brown staining). Scale bar  =  5000 µm B,C: Higher magnification photomicrographs show expression of these channels at the apical membrane of the epithelium lumen. Sections were counterstained with Harris’ hematoxylin (blue staining). Negative controls were carried out through the substitution of the primary antibody with an isotype control (top panels). Scale bar  =  1000 µm

**Figure 2:**
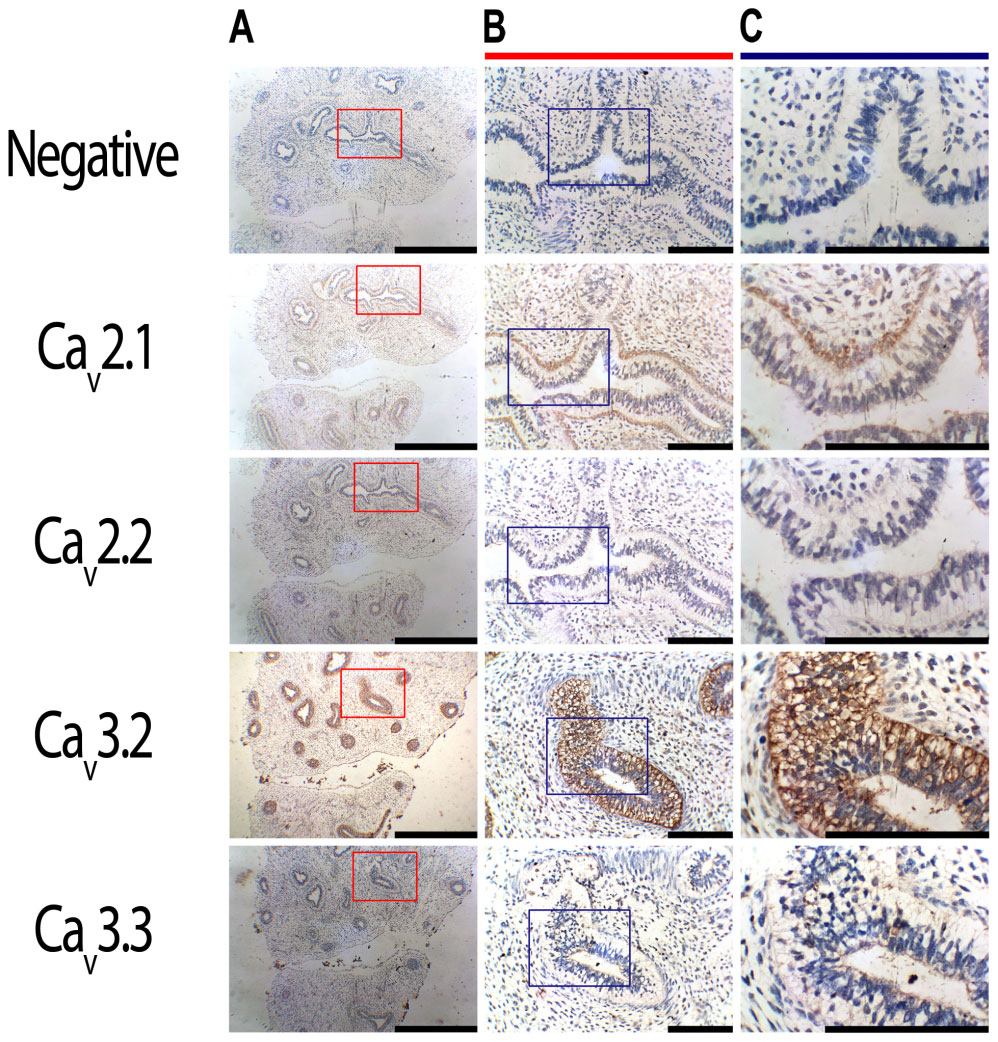
Further characterisation of the expression of voltage-gated calcium channels in the developing human lung. 5 µm-thick formalin-fixed, paraffin-embedded serial sections of 11 week post-conception human fetal lungs were dewaxed and used for immunohistochemistry. **A:** Expression of P/Q type, Ca_v_2.1, and of T-type, Ca_v_3.2, calcium channels could be detected at the basolateral side of epithelial cells and in smooth muscle cells, visualised using DAB (brown staining). Scale bar  =  5000 µm. **B,C:** Higher magnification images (40x and 100x) show little-to-no expression of the N-type calcium channel, Ca_v_2.2 or the T-type, Ca_v_3.3 in the lung parenchyma. Negative controls were carried out through the substitution of the primary antibody with an isotype control. Sections were counterstained with Harris’ hematoxylin (blue staining). Scale bar  =  1000 µm.

**Figure 3:**
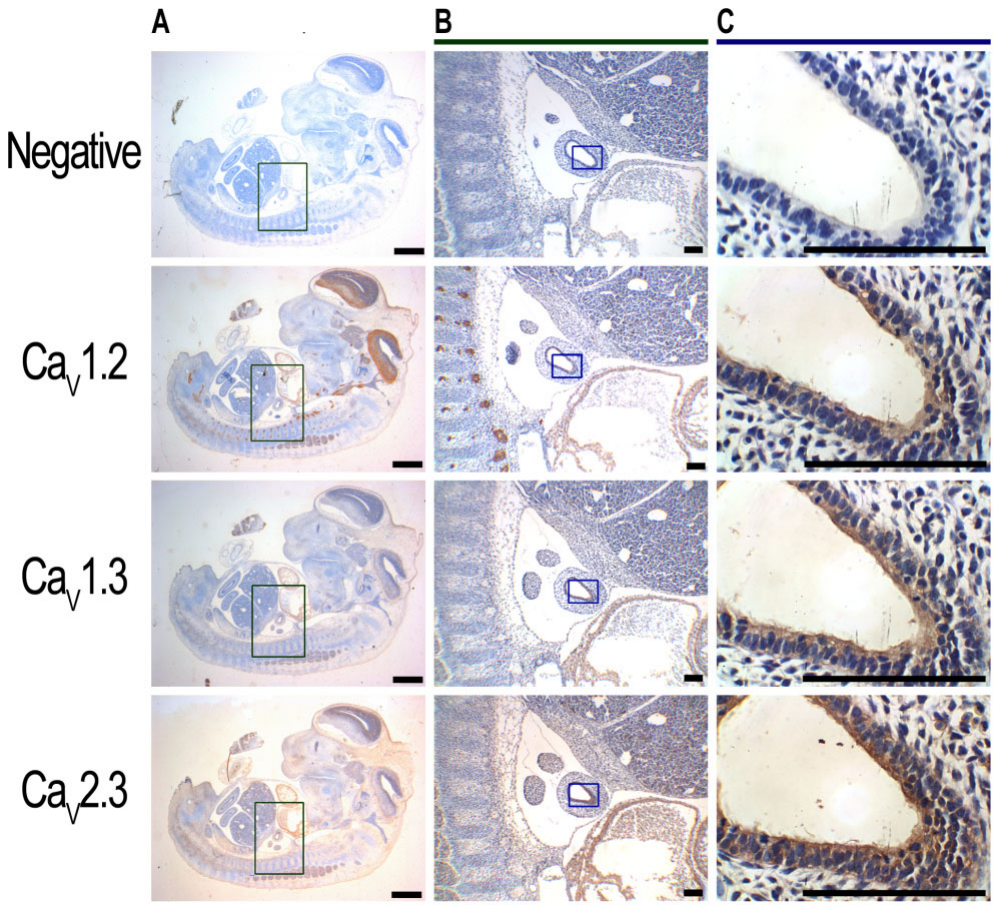
The L-type calcium channels, Ca_v_1.2 and Ca_v_1.3, and the R-type calcium channel, Ca_v_2.3, are expressed in the epithelium of E12.5 mouse lungs. A: Immunohistochemistry carried out on 5 µm-thick, paraffin-embedded serial sections of C57BL/6 E12.5 whole embryos shows expression of Ca_v_1.2, Ca_v_1.3 and Ca_v_2.3 in the heart (all), neuronal outgrowths on the spinal cord (Ca_v_1.2) and CNS (Ca_v_1.2 and Ca_v_2.3) (brown staining). Scale bar  =  50 µm. B, C: Higher magnification images demonstrate that Ca_v_1.2, Ca_v_1.3 and Ca_v_2.3 are also expressed apically in the epithelium of the developing lung. Sections were counterstained with Harris’ haematoxylin (blue staining). Negative controls were performed by substitution of the primary antibody with an isotype control (A-C, top panels). Scale bar  =  1000 µm.

Immunohistochemistry on serial sections of human fetal lungs show that the airway epithelium expressed Ca_v_1.2, Ca_v_1.3 and Ca_v_2.3 at the apical membrane (Figure 1), with little or no expression of Ca_v_2.2 and Ca_v_3.3 (Figure 2). Ca_v_2.1 and Ca_v_3.2 were also observed at the basolateral membrane of the airway epithelium and in the smooth muscle (Figure 2). A summary of the types of VGCC found in the human lung, their cellular distribution and the specific inhibitors is reported in Table 1. In the mouse, Ca_v_1.2 (cardiac L-type) was expressed in the developing heart, brain and neuronal outgrowths on the spinal cord [26] (Figure 3A and 3B). Ca_V_1.3 (neuronal L-type) and Ca_v_2.3 (R-type) expression were detected within mouse brain [27], [28] and heart [29], [30] (Figure 3) and in the cochlea [31] (Ca_v_1.3, Figure S1). Consistent with what was observed in the human, expression of all three of these channels was also detected in the apical membrane of the airway epithelium of the pseudoglandular mouse lung (Figure 3B and 3C). A summary of the expression of the different types of VGCC in the human lung is presented in table 1.

**Table 1:** Summary of the expression of VGCC in the pseudoglandular human lung.

	Epithelium (apical)	Epithelium (basolateral)	Smooth muscle	Blocker
Ca_v_1.2 (L-type, A1C)	Y	Y	Y	Nifedipine
Ca_v_1.3­ (L-type, A1D)	Y	N	N	Nifedipine
Ca_v_2.1 (P/Q-type, A1A)	N	N	Y	Agatoxin
Ca_v_2.2 (N-type, A1B)	N	N	N	ω-Conotoxin
Ca_v_2.3 (R-type, A1E)	Y	N	N	SNX-482
Ca_v_3.2 (T-type, A1H)	N	Y	Y	None
Ca_v_3.3 (T-type, A1I)	N	N	N	None

### Epithelial voltage changes occur within the epithelium of the pseudoglandular mouse lung

**Figure 4:**
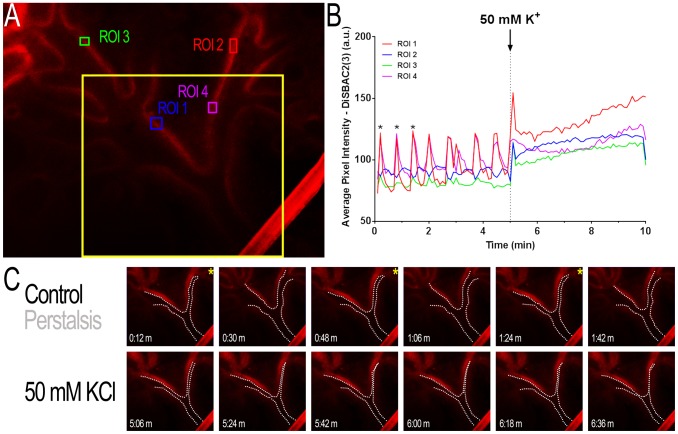
Peristaltic contractions lead to increases in voltage-dependent fluorescence in the epithelium of the developing lung. Lung explants from E12.5 C57BL/6 mice were cultured for 48 hours before being loaded with DiSBAC_2_(3) through intra-luminal injection. A: a lung successfully loaded with DiSBAC_2_(3) with regions of interest (ROI) for the graph in B. The yellow square demonstrates the area showed in C. B: changes in DiSBAC_2_(3) fluorescence intensity (arbitrary units, a.u.) for the ROIs shown in A, with increases in DiSBAC_2_(3) fluorescence intensity indicative of membrane depolarization. The dotted line shows that airway occlusion, evoked by 50 mM KCl, is accompanied by a slow but sustained depolarization of the membrane. Asterisks show spikes in fluorescence intensity, which correlate with contraction of the lungs during peristalsis, as seen in C. C: zoomed-in area of the lung, as shown by the yellow square in A. White dotted lines highlight the apparent movements of the luminal area during normal airway peristalsis (control) or after the addition of 50 mM KCl. Note that yellow asterisks correlate with lung airway contraction and an apparent spike in fluorescence intensity (also highlighted by black asterisks in B), suggesting that changes in epithelium membrane polarization occurs during peristalsis.

In order to understand why VGCC are expressed in the fetal lung epithelium and how they might exert a physiological effect, the epithelial layer of the explants was loaded with a voltage-sensitive dye, DiSBAC_2_(3). This dye was injected directly into the lung lumen, and this manoeuvre resulted in its exclusive loading into the epithelial sheet lining the developing airway. Confocal imaging of DiSBAC_2_(3) fluorescence in the focal plane of the epithelium showed robust and reproducible voltage oscillations, which coincided with the fetal airway peristalsis (Figure 4, Video S1). 

### The L- and R-type Ca^2+^ channel blockers, nifedipine and SNX-482, rescue the inhibitory effects of fetal hypercalcaemia on lung branching morphogenesis

**Figure 5:**
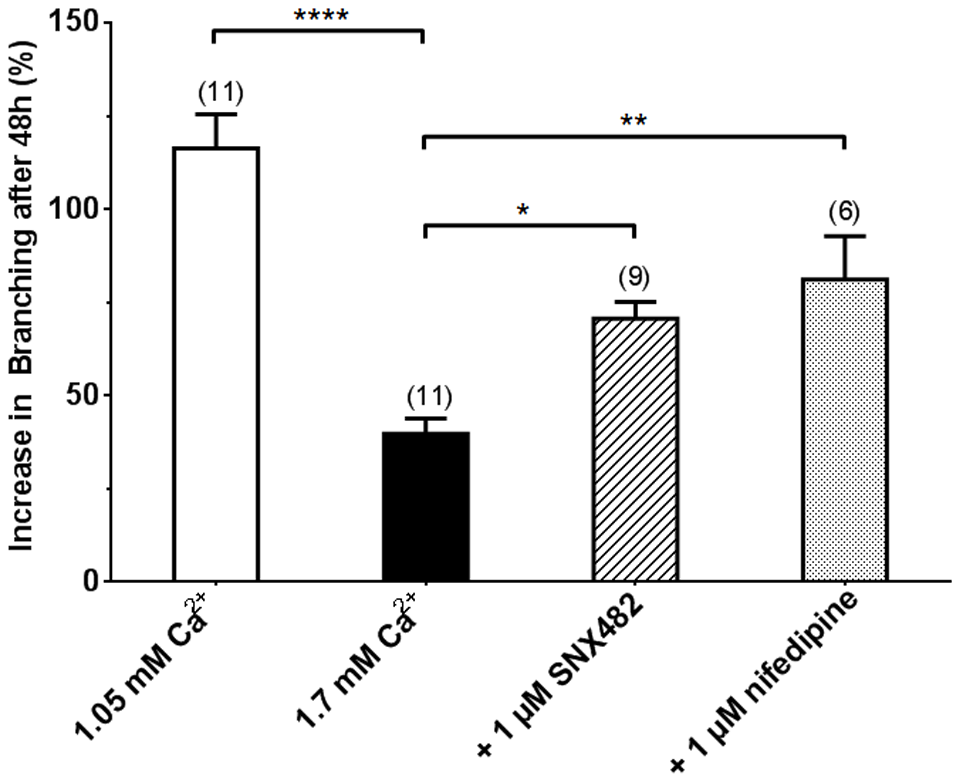
L-type/R-Type Ca^2+^_o_ channel blockers rescue Ca^2+^_o_-dependent inhibition of lung branching morphogenesis. Effect of the calcium channel blockers, SNX-482 and nifedipine, in the presence of 1.70 mM Ca^2+^_o_ on lung branching morphogenesis in C57BL/6 E12.5 mouse lungs after 48 h in culture. Lungs cultured in presence of 1 µM SNX-482 and 1 µM nifedipine showed significant rescue of Ca^2+^-dependent suppression of branching compared to those cultured in medium containing 1.70 mM Ca^2+^_o_ alone. Data were pooled from 3 – 4 separate isolations for each condition and are presented as mean ± SEM. *, p<0.05; **, p<0.01; ***, p<0.001.

To demonstrate that calcium influx through VGCC is directly responsible for the inhibitory effects of fetal hypercalcaemia on lung branching morphogenesis, we tested the effects of pharmacological blockers of L-type (Ca_V_1.2 and 1.3) or R-type (Ca_V_2.3) calcium channels on branching morphogenesis. Pseudoglandular mouse lung rudiments were cultured for 48 h in medium containing 1.7 mM Ca^2+^_o_ in the presence of either SNX-482 or nifedipine (1 uM for both), and branching morphogenesis was assessed after 48h. Paired vehicle controls were carried out in the presence of 0.001% (v/v) ethanol (for nifedipine) or 0.001% (v/v) DMSO (for SNX-482). Neither ethanol (not shown) nor DMSO [12] affected branching morphogenesis and data obtained from these controls were pooled. In accordance with our previously published results, culturing E12.5 C57BL/6 mouse lung explants in fetal hypercalcemic conditions (1.7 mM Ca^2+^_o_) led to a reduction in the number of terminal lung branches, compared to those in 1.05 mM Ca^2+^_o_ from 116.2±9.15 to 39.8±4.1 (n = 11, p<0.001). Addition of nifedipine, presumably acting at both the epithelial and smooth muscle L type Ca^2+^ channels, substantially rescued the inhibitory increase of the high Ca^2+^ so that branching in E12.5 mouse lungs cultured in the presence of 1.7 mM Ca^2+­­^_o_ and 1 µM nifedipine was 81.1±11.7% (n = 6, p<0.01. Figure 5).

Importantly, 1 µM SNX-482, which can only inhibit the Ca_v_2.3 channel in the epithelium, also partially rescued the suppressive effect of high Ca^2+^. Thus, airway branching increased from 39.8±4.1% to 70.6±4.5% (n = 9, p<0.05. Figure 5). These observations indicate that: i) blocking Ca^2+^_o_ influx through VGCC partly rescues Ca^2+^_o_-dependent inhibition of branching morphogenesis, and that; ii) the hypercalcaemic suppression of branching is due, in part, to calcium influx through R-type channels, expressed exclusively in the apical membrane of the airway epithelium.

## Discussion

Fetal development occurs in a hypercalcaemic environment, compared to that of the adult. We have demonstrated previously that this relative fetal hypercalcemia is an important signal in the pseudoglandular lung, balancing branching morphogenesis with fluid secretion via developmentally regulated expression of the CaSR [12], [14]. In addition to the CaSR, a functional role for VGCC has been postulated in the developing lung. The existence of L-type Ca^2+^ channels in the fetal mouse lung has been previously hypothesised by Roman who showed that treatment of pseudoglandular lung rudiments with nifedipine led to the formation of hypoplastic lungs [19]. Because nifedipine blocks voltage-gated Ca^2+^ channels, these effects have been ascribed to loss of the spontaneous airway peristalsis, which would occur as a consequence of its inhibition of voltage-gated Ca^2+^ channels in the parabronchial smooth muscle cells surrounding the developing airways [19], [32]. However, evidence that nifedipine treatment does not affect cleft formation between branch points [19], together with the fact that branching morphogenesis precedes the development of airway contractions [19] seem to rule out an exclusive role of airway peristalsis in branching morphogenesis. To evaluate the contribution that VGCC make to lung development, we assessed the functional expression of a variety of VGCC in the developing human and mouse lung, and the impact of their pharmacological manipulation on branching morphogenesis.

Our data show that several members of the Ca_v_ family are highly expressed in pseudoglandular mouse and human lung not only in the smooth muscle, but surprisingly also within the epithelium of the developing human and mouse lung. Specifically, we demonstrated significant expression of the L-type calcium channels, Ca_v_1.2 and Ca_v_1.3, and also the R-type calcium channel, Ca_v_2.3, in the lung epithelium. In addition, the human fetal lung epithelium exhibits abundant expression of the P/Q-type Ca_v_2.1 and of the T-type Ca_v_3.2. Although mostly expressed in cardiac, neuronal and endocrine cells, VGCC expression has been previously reported in a limited number of epithelial cells of the postnatal lung, with Ca_v_2.3 being expressed in Clara-like cells and airway smooth muscle bundles, and Ca_v_1.3 expressed in the apical membranes of a small number of epithelial cells [22]. Ca_v_3.2 expression has also been previously described in other non-excitable tissues, namely the adult human kidney and lung endothelium [22], [33]. However, this is the first study to report on the expression of such a range of voltage-gated Ca^2+^ channels in the embryonic lung, many of which are expressed within the epithelium. Since Ca_v_1.2 is absent from the postnatal mouse lung [22], our study suggests that, similarly to what we observed previously for the CaSR [12], Ca_v_1.2 expression is also developmentally regulated and confined to the fetal lung, suggesting that this channel plays a role in embryonic lung development. Recent evidence suggests that single nucleotide polymorphisms in Ca_v_1.2 are associated with a range of psychiatric disorders, including autism spectrum disorders [34]. Interestingly, a study carried out in 459 subjects revealed that, of the 49 of these who exhibited defective branching (*i.e*., “doublets”) in the lower airway, all of them had autism spectrum disorders. Indeed, the authors have proposed airway doublets as markers for autism [35].

Having ascertained the expression of VGCC in the pseudoglandular human and mouse lungs, we set out to determine their contribution to lung development. To dissect peristalsis-driven events from epithelial Ca^2+^ channel-mediated effects, we used inhibitors of VGCC present in both smooth muscle and epithelium (namely Ca_v_1.2 and 1.3), *i.e*., nifedipine, and a specific blocker of channels expressed solely within the epithelium (namely Ca_v_2.3), *i.e*., SNX-482. Our observations show that nifedipine rescues the inhibitory effects of fetal hypercalcemia on lung branching morphogenesis to a level, which is comparable to that seen in the presence of medium containing the lower [Ca^2+^]_o_ [12], suggesting that Ca^2+^_o_ influx through VGCC contributes to the hypercalcaemic suppression of branching. More importantly SNX-482 also rescues the inhibitory effects of fetal hypercalcaemia on branching morphogenesis. SNX-482 is a specific blocker of Ca_v_2.3 present exclusively in the developing human and mouse lung epithelium, and therefore ineffective at blocking airway peristalsis. Therefore, our results suggest that airway peristalsis contributes to, but cannot fully account for, the Ca^2+^-dependent regulation of branching morphogenesis.

Finally, to assess whether the epithelial VGCC can be activated by a depolarising stimulus, we measured changes in membrane potential in cells lining the lumen of the developing mouse bronchi by injecting the voltage-sensitive fluorescent dye DiSBAC_2_(3) directly into the lung lumen 30–45 min before experimenting. DiSBAC_2_(3) is a membrane potential dye, which enters the cell upon depolarisation where it binds to intracellular proteins and, as such, unlikely to leak out the cell from the basolateral side into the mesenchyme. Our observations show that, in fetal hypercalcemic conditions, rhythmic depolarisation and hyperpolarisation can be observed, which coincide with airway peristalsis, as shown by cyclic occlusion of the lung lumen. Airway peristalsis is initiated by spontaneous smooth muscle cell contractions in the pacemaker regions of the proximal airway [9]. It produces a pulsatile wave of fluid towards the tip of the growing lung, and is thought to be essential to lung growth, as shown by experiments demonstrating that manoeuvres aimed at inhibiting or accelerating airway peristalsis result in impaired or increased lung growth *in vitro* [1], [9]. Our study suggests that peristalsis-driven cycles of depolarisation and hyperpolarisation can also lead to activation of epithelial voltage-gated calcium channels. This mechanism would allow for branching morphogenesis to proceed in a synchronous manner with the airway expansion driven by the mechanical stimulus provided by fluid being propelled within the airway lumen.

In conclusion, ambient fetal calcium plays a crucial role in lung development. Hypercalcaemic conditions present within the fetus suppress branching morphogenesis by acting on proteins whose expression is developmentally confined to the fetal lung, namely the CaSR and VGCC. Whether the consequence of CaSR activation, or influx via VGCC, ultimately increases in [Ca^2+^]_i_ are likely to provide the underpinning stimulus for the suppressive effects of fetal hypercalcemia on branching morphogenesis. Sub-optimal lung development could result from alterations in fetal [Ca^2+^]_o_ and therefore predispose to pathological conditions later on in life, such as interstitial lung disease, via the CaSR[36], or to a reduction in airway diameter and branching defects, yielding impaired airway and secretion clearance, via VGCC [34].

## Supporting Information

Figure S1 **Expression of Ca_v_1.3 in sensory cells in the developing mouse.** Immunohistochemistry carried out on 5 µm-thick, paraffin-embedded serial sections of C57BL/6 E12.5 whole mouse embryos shows expression of Ca_v_1.3 in sensory cells (photoreceptors and cochlear cells, arrows) and neuronal outgrowths of the spinal cord (brown staining). Scale bar  =  1000 µm.
(TIF)

Video S1 **Time-lapse video of DiSBAC_2_(3) loaded lung epithelium.** A lung explant from an E12.5 C57BL/6 mouse was cultured for 48 h before being loaded with DiSBAC_2_(3) through intra-luminal injection. DiSBAC_2_(3) fluorescence was visualized using a Cell Map IC confocal laser-scanning microscope with a water-immersion BX50WI and a UM Plan Fl 10x/0.30 W objective. Lungs were secured using a slice anchor in a 35 mm cell culture dish in 5 mL of solution containing in (mM): 135 NaCl, 5 KCl, 1.23 MgCl_2_, 1.0 mM CaCl_2_, 5 HEPES, 10 glucose, pH 7.4. DiSBAC_2_(3) was excited at 532 nm and images were collected using a 560 nm long pass filter, by the direct method. Images were taken at 6s intervals over a ten minute time period at room temperature and after 5 minutes, the KCl concentration in the extracellular solution was increased to 50 mM.
(AVI)
