# Supplementary figures and images for: Fetal Calcium Regulates Branching Morphogenesis in the Developing Human and Mouse Lung: Involvement of Voltage-Gated Calcium Channels
Source: PLoS One. 2013 Nov 25;8(11):e80294. doi: 10.1371/journal.pone.0080294 (PMC3840017; doi:10.1371/journal.pone.0080294)

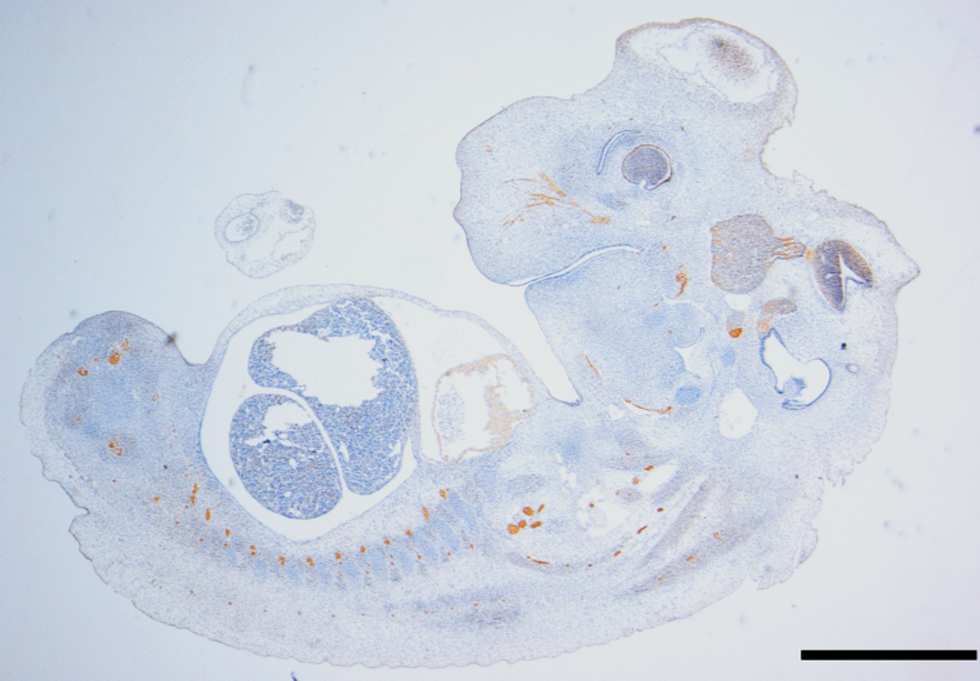

Supplement: Figure S1 — Expression of Cav1.3 in sensory cells in the developing mouse. Immunohistochemistry carried out on 5 µm-thick, paraffin-embedded serial sections of C57BL/6 E12.5 whole mouse embryos shows expression of Cav1.3 in sensory cells (photoreceptors and cochlear cells, arrows) and neuronal outgrowths of the spinal cord (brown staining). Scale bar = 1000 µm. (TIF) [file pone.0080294.s001.tif]
